# Comparison of redox and ligand binding behaviour of yeast and bovine cytochrome *c* oxidases using FTIR spectroscopy^☆^

**DOI:** 10.1016/j.bbabio.2018.05.018

**Published:** 2018-09

**Authors:** Amandine Maréchal, Andrew M. Hartley, Thomas P. Warelow, Brigitte Meunier, Peter R. Rich

**Affiliations:** a Department of Biological Sciences, Birkbeck, University of London, Malet Street, London WC1E 7HX, UK; b Department of Structural and Molecular Biology, University College London, Gower Street, London WC1E 6BT, UK; c Institute for Integrative Biology of the Cell (I2BC), CEA, CNRS, Université Paris-Sud, Université Paris-Saclay, 91198 Gif-sur-Yvette, France

**Keywords:** ATR, attenuated total reflectance, BNC, binuclear centre, C*c*O, cytochrome *c* oxidase, FTIR, Fourier transform infrared, HCO, haem‑copper oxidase, 6H-WT, yeast C*c*O with a six-histidine tag on nuclear-encoded Cox13 subunit, 6H-E243D_I_, 6H-I67N_I_ or 6H-S52D_I_, the 6H-WT strain with an additional mutation of E243D, I67N or S52D in mtDNA-encoded subunit I, Cytochrome *c* oxidase, Mitochondria, Infrared spectroscopy, Oxidoreduction, Site-directed mutagenesis, Carboxyl groups

## Abstract

Redox and CO photolysis FTIR spectra of yeast cytochrome *c* oxidase WT and mutants are compared to those from bovine and *P. denitrificans* C*c*Os in order to establish common functional features. All display changes that can be assigned to their E242 (bovine numbering) equivalent and to weakly H-bonded water molecules. The additional redox-sensitive band reported at 1736 cm^−1^ in bovine C*c*O and previously assigned to D51 is absent from yeast C*c*O and couldn't be restored by introduction of a D residue at the equivalent position of the yeast protein. Redox spectra of yeast C*c*O also show much smaller changes in the amide I region, which may relate to structural differences in the region around D51 and the subunit I/II interface.

## Highlights

• Redox-induced FTIR difference spectra of WT and mutant yeast C*c*O are presented.
• Functionally-relevant features are compared with other A1-type haem copper oxidases.
• On oxidoreduction, all show perturbations of bovine residue E242
• Introduction of bovine D51 in yeast doesn't result in an additional IR redox band.
• On photolysis of the FR-CO form all show perturbations of E242 and water molecules

## 1 Introduction

Mitochondrial cytochrome *c* oxidase (C*c*O) is a member of the A1 branch of the haem‑copper oxidase (HCO) superfamily of respiratory oxidases that catalyse the four electron reduction of molecular oxygen to water [[1], [2], [3]], conserving the free energy in the transmembrane proton electrochemical gradient. In mammals, each monomer has at least 13 different subunits (~204 kDa) and may form dimers or integrate into supercomplexes with other respiratory complexes in the inner mitochondrial membrane [4]. Subunits I, II and III are encoded by mitochondrial DNA and form the catalytic core. The remaining ‘supernumerary’ subunits, several of which can occur in tissue-specific isoforms, are nuclear DNA-encoded; their functions are receiving increasing interest with possible roles in assembly, stability, allosteric control [5] and supercomplex formation [6].

Particularly in mammalian mitochondrial C*c*O, the detailed atomic mechanism by which proton and electron transfers are coupled during catalysis remains controversial [[7], [8], [9]]. Electrons are provided one at a time by reduced cytochrome *c* in the intermembrane space to a dicopper centre (Cu_A_) within subunit II. They are then transferred through subunit I *via* a *bis*-histidine coordinated A-type haem (haem *a*) to the O_2_ reducing binuclear centre (BNC), composed of another A-type haem (haem *a*_3_) and a copper atom (Cu_B_). A large body of experimental data indicates that each electron transfer into the BNC is coupled to the uptake of two protons from the mitochondrial matrix: one pumped proton, ultimately translocated across the membrane, and one substrate proton, directed to the O_2_ reduction site [10]. These protons must traverse the interior of the largely hydrophobic enzyme along hydrophilic channels formed by arrays of protonatable/polar residues and associated water molecules [[11], [12], [13]]. Three candidate structures, the D, K and H channels, have been identified within subunit I from the X-ray derived atomic structures of both mitochondrial (bovine) and bacterial A1-type HCOs [14,15]. In bacterial A1-type HCOs, a large body of kinetic and mutant data indicates that the D and the K channels provide the route for substrate protons at different stages of the oxygen reduction cycle, and that the D channel provides a part of the pathway for all pumped protons [10]. Briefly, in the most widely accepted mechanism [9,10], for each electron transfer from haem *a* into the BNC, a pumped proton is transferred from E242 (bovine numbering) at the top of the D channel *via* a gated route into a temporary proton trap in the region above the BNC, possibly close to the bound Mg^2+^ ion [16]. The opposite charges of the trap proton and the negative BNC electrostatically stabilise each other. However, reduction of the BNC creates a high pK protonatable site for a substrate proton. Protonation of this site *via* the K or D channel neutralises the BNC charge, which in turn induces release of the trap proton into the P phase *via* a route that is not yet well-defined. This same mechanism may be operative in mammalian C*c*Os, though a different mechanism involving transfer of the pumped protons *via* the H channel, rather than through the D channel, has been proposed, based on structural and more limited functional studies [8].

The D channel extends from D91 at the N phase surface to E242, located roughly equidistant from both haem edges. E242 (with equivalents of E243 in *S. cerevisiae*, E278 in *P. denitrificans* and E286 in *R. sphaeroides* C*c*Os and E286 in *E. coli bo*_3_ quinol oxidase) and the D channel are conserved throughout the A1-type HCOs [17]. Mutagenesis studies in bacterial [7,18] and yeast [19,20] systems have shown that they are essential for activity. The most recent proposal of H channel function in bovine C*c*O [21,22] includes a proton pathway from D407 in the N phase towards the edge of haem *a* that is gated by a redox- and ligand-sensitive movement of S382. When open, this pathway provides protonic connection to the path leading to the proton trap site around the bound Mg^2+^. Protons are then envisaged to be released into the P phase from this trap *via* the top section of the H channel, in a pathway gated by the redox state of haem *a* and the peptide bond between Y440 and S441, finally arriving at D51 at the P phase subunit I/II interface [23].

Fourier transform infrared (FTIR) spectroscopy has revealed functional aspects of E242 and D51 [24], particularly from the characteristic bands of their protonated forms in the relatively uncluttered 1800–1700 cm^−1^ range [25]. Here we compare new redox and CO photolysis difference FTIR data of yeast C*c*O with equivalent published spectra of bovine and bacterial C*c*Os.

## 2 Materials and methods

Yeast extract was purchased from Ohly GmbH, Germany, detergents were from Melford Laboratories, UK, Ni^2+^-affinity resin (His-bind®) from Novagen, D_2_O (D, 99.9%) from Cambridge Isotope Laboratories, Inc. All other reagents were purchased from Sigma Aldrich.

### 2.1 Mutant constructs and enzyme preparation

The addition of a 6-histidine-tag on the nuclear DNA-encoded subunit Cox13 of yeast *Saccharomyces cerevisiae* C*c*O and the subsequent construction of mutant strains with additional single point mutation of E243D, I67N or S52D in the mitochondrial DNA-encoded subunit Cox1 were described in [26]. Growth of the yeast cells in galactose medium, and the protocols for the preparation of mitochondria and the purification of the resulting 6H-WT, 6H-E243D_I_, 6H-I67N_I_ and 6H-S52D_I_ C*c*Os with n-dodecyl-β-d-maltoside by Ni^2+^-affinity and DEAE Sepharose CL-6B ion exchange chromatography were as in [19,26]. WT *P. denitrificans* C*c*O was provided by Mårten Wikström (University of Helsinki, Finland) and was prepared as in [27].

### 2.2 ‘ATR-ready’ protein sample preparation and electrochemically induced ATR-FTIR difference spectroscopy

Depletion of detergent for the preparation of ‘ATR-ready’ protein samples was performed from 250 pmol of purified yeast 6H-WT or mutated C*c*O as described in [28]. The resulting ‘ATR-ready’ C*c*O was immediately placed on a 3-reflection silicon attenuated total reflectance (ATR) prism (DuraSampI*IR* II, SensIR/Smith Detection). After drying with a gentle stream of N_2_ gas, the protein film was rehydrated with a buffer of 100 mM K-phosphate, 100 mM KCl at pH 6.0. The same procedure was followed for analyses of H/D exchange effects except that all solutions were prepared with D_2_O and at pD 6.0. pD values were adjusted with a standard glass pH electrode assuming pD = pH (meter reading) + 0.4 [29].

An electrochemical cell built in-house that allows simultaneous recording of UV/visible and IR absorption spectra [30] was assembled on top of the protein film. The working electrode was a platinum grid held approximately 0.2 mm above the protein film. A platinum sheet counter electrode and Ag/AgCl/KCl reference electrode in 100 mM K-phosphate, 100 mM KCl at pH/pD 6.0 were connected to the sample chamber by a Vycor frit [31]. The sample chamber was filled with the same solution containing in addition 50 μM anthraquinone-2-sulfonate (E_m,7_ = −225 mV) and 1 mM ferricyanide (E_m,7_ = 430 mV) as redox mediators. All redox potentials are quoted *versus* the standard hydrogen electrode.

Reduced *minus* oxidised mid-IR difference spectra were recorded at 4 cm^−1^ resolution at room temperature in ATR mode on a Bruker IFS 66/S spectrometer equipped with a liquid nitrogen-cooled MCT-A detector. All frequencies cited have an accuracy of ±1 cm^−1^. For each IR spectrum recorded, 500 interferograms were averaged before Fourier transformation. Redox transitions were induced by applying potentials of −350 mV/+500 mV (reduction/oxidation) with a potentiostat (Princeton Applied Research) following the protocol described in [31] for a typical redox cycle. Equilibration times for stabilisation of all UV/visible and IR absorbance changes were typically 12 min in both directions. Redox cycles were repeated to improve signal to noise depending on the IR signal size and stability of the protein film. Spectra presented are averages of data from at least two distinct samples and the number of redox cycles used to produce each IR spectrum is given in figure legends.

### 2.3 Light induced CO photolysis FTIR difference spectroscopy

The preparation of fully reduced CO bound samples of yeast and *P. denitrificans* C*c*Os and recording of light *minus* dark mid-IR difference spectra in transmission mode was as described in [19]. The data were acquired at 4 cm^−1^ resolution on a Bruker Vertex 80v spectrometer equipped with a liquid nitrogen-cooled MCT-C detector with the optics compartment kept under vacuum (<2 hPa). A narrow band filter (Northumbria Optical Coatings Ltd.) was used to isolate the high-frequency region (3800–3600 cm^−1^) and increase signal/noise [32].

### 2.4 Spectra correction and data treatment

When necessary, IR spectra were corrected for the contribution of water vapour, redox mediators, pH change and total protein or lipid changes due to a slight swelling of the protein film on recording in ATR mode. This was done with OPUS 6.5 software (Bruker) by iterative subtraction of model spectra recorded under the conditions of the experiments. All figures were subsequently produced using OriginPro 2015 (OriginLab Corporation).

## 3 Results and discussion

### 3.1 Comparison of redox-induced FTIR spectra

**Fig. 1:**
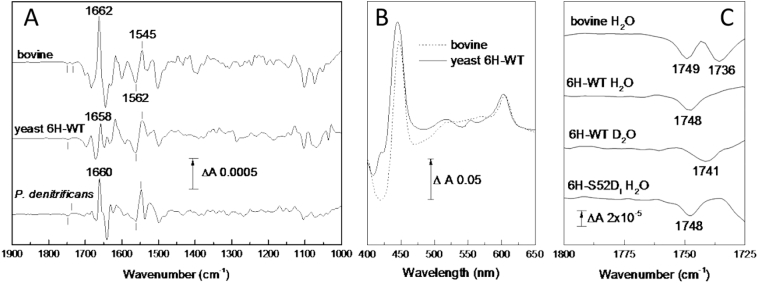
Electrochemically induced reduced *minus* oxidised ATR-FTIR difference spectra of bovine, yeast 6H-WT and *P. denitrificans* A1-type oxidases. A. The spectra of bovine (top) and yeast 6H-WT C*c*Os (middle trace) are the average of 120 redox cycles recorded at pH 8.5 and 6.0, respectively. The spectrum of *P. denitrificans* C*c*O (bottom trace) is the average of 25 redox cycles at pH 8.5. All were scaled on their amide II band change at 1562(−)/1545(+) cm^−1^. B. Typical reduced *minus* oxidised visible absorption difference spectra of bovine (dashed) and yeast 6H-WT (solid line) recorded simultaneously with IR recordings. C. Expansion of the 1800–1725 cm^−1^ region and effects of H/D exchange (labelled D_2_O, average of 190 redox cycles at pD 6.0) and S52D mutation on the yeast 6H-WT redox spectrum.

Fig. 1A presents an electrochemically-induced reduced *minus* oxidised ATR-FTIR difference spectrum of yeast 6H-WT C*c*O recorded at pH 6.0 in the mid-IR ‘fingerprint’ region (middle trace). This spectrum arises from band changes of IR-active groups that are sensitive to redox changes of the enzyme in its unligated state. Typical redox spectra of bovine mitochondrial and *P. denitrificans* A1-type enzymes are also displayed for comparison (Fig. 1A, top and bottom traces, respectively; similar data have been published in [32,33]). All spectra have been scaled on their amide II feature at 1562(−)/1545(+) cm^−1^. Major features of the spectra are similar and tentative assignments have been made for many bands (for a recent review see [31] and references therein).

**Fig. 2:**
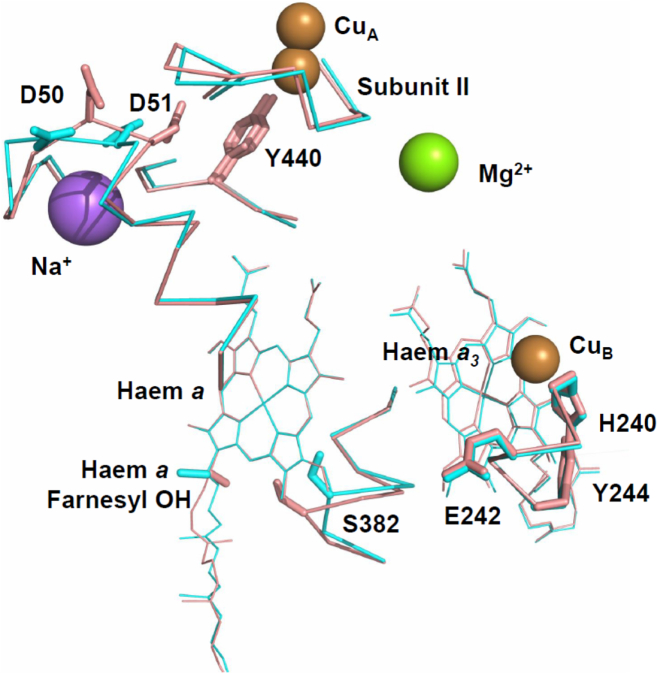
Structural comparison of bovine cytochrome *c* oxidase in oxidised and reduced states. Figure was composed using coordinates from PDB ID: 5B1A (oxidised) and PDB ID: 5B1B (reduced) structures [21] after alignment of their haem *a* moieties. Selected stretches of polypeptide backbones, amino acids and the haem *a* farnesyl hydroxyl group are shown in pink (oxidised subunit I, PDB ID: 5B1A) and cyan (reduced subunit I, PDB ID: 5B1B).

However, one difference resides in the extent of their amide I band changes in the 1650 cm^−1^ region. In bovine and *P. denitrificans* C*c*Os, a prominent peak at 1662–1660 cm^−1^ dominates the redox spectrum and has been linked to the redox changes of Cu_A_/haem *a* [31,34]. It has an intensity 2–3 times greater than their amide II changes. In yeast C*c*O, the amide I peak at 1658 cm^−1^ relative to the amide II change is much smaller. Visible absorption spectra recorded simultaneously with IR spectra (Fig. 1B) indicated that the Soret and α-band features had fully formed and were at a ratio consistent with reduction of both haems [35]; hence, the smaller amide I band does not appear to arise from incomplete redox cycling. Isotope labelling of *P. denitrificans* C*c*O has shown that amide bonds of histidine and tyrosine are major contributors to this band [33]. Comparisons of the high resolution X-ray structures for bovine [21,23] and *R. sphaeroides* [36,37] C*c*Os in their fully oxidised and reduced states have revealed three regions of redox-induced structural flexibility (Fig. 2). In *R. sphaeroides* significant changes occur in the region of the BNC associated with movements of haem *a*_3_, helix VIII, water molecules at the top of the D- and the K-channels and an H-bond between Y244 of the HPEVY pentapeptide and the haem *a*_3_ hydroxyfarnesyl [36]. Equivalent redox-induced structural changes have not been observed in all bovine C*c*O structures, despite their similar amino acid composition, and some interesting suggestions have been made as to why those changes might not have been consistently evident (see discussion in [37]). In contrast, in both *R. sphaeroides* and bovine C*c*Os changes are observed around S382, including reorientation of its side chain, though in bovine structures this also includes reorientation of part of the haem *a* hydroxyfarnesyl chain (Fig. 2). Amino acids in those two regions are conserved in yeast C*c*O and so might be expected to contribute similar amide I changes in all redox spectra, though this will require structural confirmation. A third region of redox-induced structural flexibility has been observed around residues D50/D51 in bovine C*c*O and the change of D51 in particular has been proposed to have significant functional importance [23]. D51 and its adjacent amino acids are not conserved at all in bacterial C*c*Os and no redox-induced structural changes in the equivalent region have been observed. The region in both cases is close to the subunit I/II interface, which is formed also in part from a short sequence that includes Y440 and two arginines (R438 and R439) that interact with haem propionates. The C=O amide of Y440 interacts with the D-ring propionate of haem *a via* a bridging water and its head group projects towards Cu_A_ in subunit II. Y440 is conserved in *P. denitrificans* and *R. sphaeroides* C*c*Os, but is an isoleucine in yeast C*c*O. This replacement could decrease the polarity, and hence IR intensity, of its amide I band. Studies of further amino acid replacements may help to resolve this issue.

A second clear difference is in the 1800–1725 cm^−1^ spectral region, expanded in Fig. 1C, where only protonated carboxyl groups and lipids of the samples absorb. The yeast enzyme shows a single trough at 1748 cm^−1^, as do bacterial A1-type C*c*Os at 1746 or 1745 cm^−1^ [38,39], whereas two troughs have consistently been reported in bovine C*c*O, independent of pH, at 1749 and 1736 cm^−1^ (Fig. 1C, two top traces). These bands have also been associated with reduction of Cu_A_/haem *a* [24,31,34]. The 1736 cm^−1^ feature has been assigned to D51 [24], consistent with the absence of the band and this residue in both bacterial and yeast C*c*Os ([40] and Fig. 1). Based on structural data, it was interpreted as arising from deprotonation of D51 in reduced C*c*O and was proposed to be a key function in the alternative H-channel proposal [8,23]. Direct evidence for protonated D51 in oxidised C*c*O is yet to be produced. A sufficiently high pK is difficult to reconcile with its position at the top of a helix with nearby waters and polar residues, or with the known E_m_/pH dependencies of haem *a* and Cu_A_ [[41], [42], [43]]. A 3D model of yeast C*c*O built by sequence homology onto the bovine X-ray structure shows a good alignment of features in that region, including the short helix that extends from the haem *a* histidine ligand (bovine H61, yeast H62) to the yeast equivalent of D51 (S52) [44]. Based on this, we have introduced a D residue in yeast C*c*O at the S52 locus. The mutant (6H-S52D_I_) cells had a WT growth phenotype [26] and the O_2_-reduction rate measured on mitochondrial membrane preparation indicated WT C*c*O activity at 1200 e·s^−1^ (measured as in [19]). As shown in Fig. 1C, lower trace, the mutation did not result in an additional redox-induced carboxylic acid IR signal equivalent to that in bovine C*c*O. Hence, if the bovine CcO band is indeed due to protonated D51, the data suggest that additional structural differences between bovine and yeast C*c*Os govern the band change in this region. For example, the H-bonding partners of bovine D51 (subunit I S441 and subunit II S205) that are proposed to create the protonating environment of D51 are replaced by proline and alanine, respectively, in yeast. However, at this stage assignment of the 1736 cm^−1^ signal in the bovine spectrum to another buried carboxyl group [40] or to a lipid molecule cannot be definitively ruled out.

### 3.2 Effect of mutations in yeast

The trough at 1749–1748 cm^−1^ in bovine and yeast C*c*Os (Fig. 1C, top two traces) is consistent with the same band of protonated E242 (E243 in yeast) observed in their CO photolysis spectra [19,24,45]. On H/D exchange in both yeast (Fig. 1C, lower trace) and bovine C*c*O redox spectra [24,46] the trough has a 7 cm^−1^ downshift to 1741 cm^−1^, consistent with its carboxylic acid origin. The 1748 cm^−1^ trough reported here for the yeast enzyme seems devoid of an associated peak, and hence is most likely to arise from a decrease in polarity of the E243 carboxyl on reduction of the enzyme. The same assignment and interpretation are likely for the equivalent 1749 cm^−1^ band of bovine C*c*O.

**Fig. 3:**
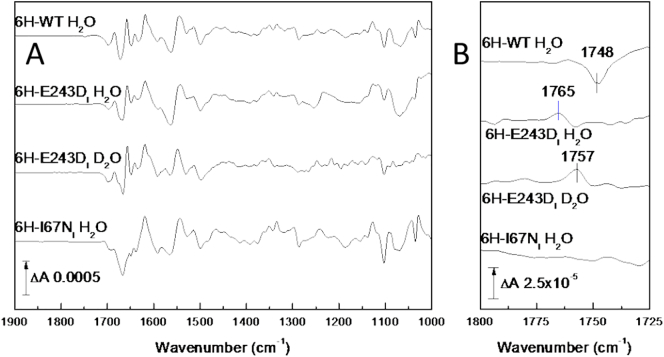
Electrochemically induced reduced *minus* oxidised ATR-FTIR difference spectra of yeast 6H-WT C*c*O and selected mutants at pH/pD 6.0. A. 1900–1000 cm^−1^ range. The 6H-WT spectrum is from Fig. 1; the 6H-E243D_I_ H_2_O and D_2_O spectra and 6H-I67N_I_ H_2_O spectrum are the average of 330, 143 and 180 redox cycles, respectively. B. Expansion of the 1800–1725 cm^−1^ spectral region.

Yeast C*c*O mutations E243D and I67N have already been reported to decrease O_2_-reduction activity with turnover numbers in mitochondria at 43% and 1% of that of 6H-WT [19,26]. Mutant 6H-E243D_I_ led to a single net positive at 1765 cm^−1^ in redox spectra, downshifted by 8 cm^−1^ to 1757 cm^−1^ in D_2_O (Fig. 3B, second and third traces from top), providing definitive assignment to E243. Equivalent E/D replacements have been made previously in bacterial C*c*Os. These resulted in the disappearance of the trough observed at 1746 cm^−1^ in WT *P. denitrificans* C*c*O [47] and of the 1745(−)/1735(+) cm^−1^ pair in WT *E. coli bo*_3_ [48] and *R. sphaeroides aa*_3_ oxidases [49]. In the latter case, a new signal from the replacement aspartic acid was observed at 1738(−)/1729(+) cm^−1^. All three studies provided definitive assignment of the redox-induced IR signals to their E243 equivalents.

Mutant 6H-I67N_I_ led to disappearance of the E243 band in redox difference spectra (Fig. 3B, lower trace), consistent with the lack of a bandshift of E243 in its CO photolysis spectra [19]. In addition, this mutation further decreased band changes in the amide I region. As discussed above (Section 3.1), the HPEVY pentapeptide is conformationally flexible in *R. sphaeroides* C*c*O [36,50]. If this is also the case in yeast C*c*O, a contribution of these residues to amide I changes in WT redox spectra, which are lost due to constraints imposed in the 6H-I67N_I_ mutant, can be suggested.

### 3.3 Water molecule rearrangements

**Fig. 4:**
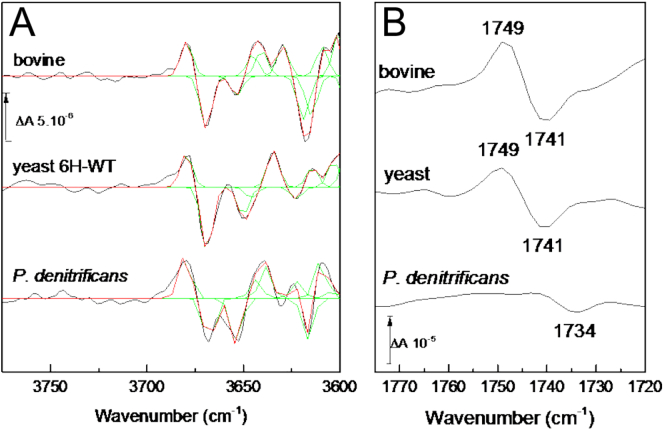
CO photolysis induced FTIR spectra. A. Water molecule rearrangements in the 3775–3600 cm^−1^ region. Yeast 6H-WT data (middle trace) are the average of 4000 light *minus* dark difference spectra. Data from bovine and *P. denitrificans* (top and lower traces) are from [32]. B. Associated protonated E242 signals in the 1775–1720 cm^−1^ region. The spectrum presented for *P. denitrificans* is the average of 2000 light *minus* dark difference spectra. All spectra are scaled on the extent of their carbon monoxide photolysis band at 1963 cm^−1^.

Water molecules are key in the transfer of both substrate and translocated protons within the channels. Their role can be structural, for instance in orienting protonatable groups, or dynamic by forming transient H-bonded networks to conduct protons in a Grotthuss mechanism. Changes have been reported in the 3700–3600 cm^−1^ range on oxidoreduction of *P. denitrificans* [51] and bovine [32] C*c*Os. More extensive changes were also reported in that region on CO photolysis from the fully reduced state of these C*c*Os [32] and were attributed to weakly H-bonded water molecule rearrangements. Fig. 4A shows equivalent data for yeast 6H-WT C*c*O (middle trace). Above 3600 cm^−1^, the difference spectrum was simulated with 8 Gaussian functions with full widths at half maximum fixed at 6 cm^−1^, similar to what was required to simulate spectra from bovine and *P. denitrificans* C*c*Os [32].

In bovine and yeast C*c*Os, signals of these weakly H-bonded waters and of protonated E242 appear in the same CO photolysis spectra (Fig. 4B, top and middle traces), suggesting a possible mechanistic connection. To date a signal from E242 equivalents has only been reported in CO photolysis spectra from the mixed valence CO forms of bacterial A1-type C*c*Os, and never from their fully reduced CO state [52,53]. However, Fig. 4B (lower trace) presents a CO photolysis spectrum of *P. denitrificans* at high signal/noise, revealing a trough at 1734 cm^−1^ with no clear positive. Hence, it is likely to arise from an extinction coefficient decrease of E278 (equivalent to bovine E242) due to a polarity decrease on CO photolysis, consistent with the wavenumber of the band attributed to E278 in the fully reduced enzyme at 1736–1734 cm^−1^ in redox-induced IR spectra [38,47]. Hence, in all C*c*Os tested to date, perturbations of water molecules and E242 can be observed.

## 4 Conclusions

Yeast is a genetically-amenable system which can be used to investigate mitochondrial C*c*Os. By comparison with bacterial and mammalian C*c*Os, important common functional features can be identified. In this report, we have discussed assignment and interpretation of published redox and CO photolysis IR spectra of bovine, yeast and *P. denitrificans* C*c*Os together with new IR redox data for yeast C*c*O (Fig. 1A and C, 6H-WT, -E243D_I_, -I67N_I_ and -S52D_I_) and new IR CO photolysis data for *P. denitrificans* (Fig. 4B) and yeast (Fig. 4A) WT C*c*Os. In both types of spectra, all C*c*Os exhibit changes of their E242 equivalent, highlighting its central mechanistic importance. Bovine C*c*O displays an additional redox sensitive band at 1736 cm^−1^ which was suggested to arise from protonated D51. This signal is absent from yeast (Fig. 1A) and bacterial C*c*Os which indeed lack D51 equivalent and are structurally different in this region. Reintroduction of D51 in yeast C*c*O (Fig. 1C) failed to induce the signal. Redox spectra of yeast C*c*O also show much smaller changes in the amide I region, which may also relate to differences in the region around D51 and the subunit I/II interface. The yeast mutation I67N appears to constrain E243 ([19] and Fig. 3) and also further decreases amide I region changes, which may be related to constraint of the HPE_243_VY pentapeptide in which E243 resides. In all three C*c*Os, CO photolysis results additionally in perturbation of signals attributable to weakly H-bonded water molecules ([32] and Fig. 4). Their consistent presence points to a functional importance but further mutation work is necessary to locate them structurally and hence establish their role.

## Transparency document

Transparency document. Image 1

## References

[1] García-Horsman J.A., Barquera B., Rumbley J., Ma J., Gennis R.B. (1994). The superfamily of heme-copper oxidases. J. Bacteriol..

[2] Hemp J., Gennis R.B., Schäfer G., Penefsky H.S. (2008). Diversity of the Heme-copper Superfamily in Archaea: Insights From Genomics and Structural Modeling.

[3] Sousa F.L., Alves R.J., Ribeiro M.A., Pereira-Leal J.B., Teixeira M., Pereira M.M. (2012). The superfamily of heme-copper oxygen reductases: types and evolutionary considerations. Biochim. Biophys. Acta.

[4] Schägger H., Pfeiffer K. (2000). Supercomplexes in the respiratory chains of yeast and mammalian mitochondria. EMBO J..

[5] Kadenbach B., Hüttemann M. (2015). The subunit composition and function of mammalian cytochrome *c* oxidase. Mitochondrion.

[6] Letts J.A., Fiedorczuk K., Sazanov L.A. (2016). The architecture of respiratory supercomplexes. Nature.

[7] Wikström M., Sharma V., Kaila V.R.I., Hosler J.P., Hummer G. (2015). New perspectives on proton pumping in cellular respiration. Chem. Rev..

[8] Yoshikawa S., Shimada A. (2015). Reaction mechanism of cytochrome c oxidase. Chem. Rev..

[9] Rich P.R. (2017). Mitochondrial cytochrome *c* oxidase: catalysis, coupling and controversies. Biochem. Soc. Trans..

[10] Wikström M., Krab K., Sharma V. (2018). Oxygen activation and energy conservation by cytochrome *c* oxidase. Chem. Rev..

[11] Nagle J.F., Tristram-Nagle S. (1983). Hydrogen bonded chain mechanisms for proton conduction and proton pumping. J. Membr. Biol..

[12] DeCoursey T.E. (2003). Voltage-gated proton channels and other proton transfer pathways. Physiol. Rev..

[13] Rich P.R., Maréchal A. (2013). Functions of the hydrophilic channels in protonmotive cytochrome *c* oxidase. J. R. Soc. Interface.

[14] Iwata S., Ostermeier C., Ludwig B., Michel H. (1995). Structure at 2.8 Å resolution of cytochrome *c* oxidase from *Paracoccus denitrificans*. Nature.

[15] Tsukihara T., Aoyama H., Yamashita E., Tomizaki T., Yamaguchi H., Shinzawa-Itoh K., Nakashima R., Yaono R., Yoshikawa S. (1996). The whole structure of the 13-subunit oxidized cytochrome *c* oxidase at 2.8 Å. Science.

[16] Kaila V.R.I., Sharma V., Wikström M. (2011). The identity of the transient proton loading site of the proton-pumping mechanism of cytochrome *c* oxidase. Biochim. Biophys. Acta.

[17] Pereira M.M., Santana M., Teixeira M. (2001). A novel scenario for the evolution of haem-copper oxygen reductases. Biochim. Biophys. Acta.

[18] Konstantinov A.A., Siletsky S., Mitchell D., Kaulen A., Gennis R.B. (1997). The roles of the two proton input channels in cytochrome *c* oxidase from *Rhodobacter sphaeroides* probed by the effects of site-directed mutations on time-resolved electrogenic intraprotein proton transfer. Proc. Natl. Acad. Sci. U. S. A..

[19] Maréchal A., Meunier B., Rich P.R. (2012). Assignment of the CO-sensitive carboxyl group in mitochondrial forms of cytochrome *c* oxidase using yeast mutants. Biochim. Biophys. Acta.

[20] Näsvik Öjemyr L., Maréchal A., Vestin H., Meunier B., Rich P.R., Brzezinski P. (2014). Reaction of wild-type and Glu243Asp variant yeast cytochrome *c* oxidase with O_2_. Biochim. Biophys. Acta.

[21] Yano N., Muramoto K., Shimada A., Takemura S., Baba J., Fujisawa H., Mochizuki M., Shinzawa-Itoh K., Yamashita E., Tsukihara T., Yoshikawa S. (2016). The Mg^2+^-containing water cluster of mammalian cytochrome c oxidase collects four pumping proton equivalents in each catalytic cycle. J. Biol. Chem..

[22] Shimada A., Kubo M., Baba S., Yamashita K., Hirata K., Ueno G., Nomura T., Kimura T., Shinzawa-Itoh K., Baba J., Hatano K., Eto Y., Miyamoto A., Murakami H., Kumasaka T., Owada S., Tono K., Yabashi M., Yamaguchi Y., Yanagisawa S., Sakaguchi M., Ogura T., Komiya R., Yan J., Yamashita E., Yamamoto M., Ago H., Yoshikawa S., Tsukihara T. (2017). A nanosecond time-resolved XFEL analysis of structural changes associated with CO release from cytochrome *c* oxidase. Sci. Adv..

[23] Yoshikawa S., Shinzawa-Itoh K., Nakashima R., Yaono R., Yamashita E., Inoue N., Yao M., Fei M.J., Libeu C.P., Mizushima T., Yamaguchi H., Tomizaki T., Tsukihara T. (1998). Redox-coupled crystal structural changes in bovine heart cytochrome *c* oxidase. Science.

[24] Okuno D., Iwase T., Shinzawa-Itoh K., Yoshikawa S., Kitagawa T. (2003). FTIR detection of protonation/deprotonation of key carboxyl side chains caused by redox change of the Cu_A_-heme *a* moiety and ligand dissociation from the heme *a*_3_-Cu_B_ center of bovine heart cytochrome *c* oxidase. J. Am. Chem. Soc..

[25] Rich P.R., Iwaki M., Wikström M. (2005). Biophysical and Structural Aspects of Bioenergetics.

[26] Meunier B., Maréchal A., Rich P.R. (2012). Construction of histidine-tagged yeast mitochondrial cytochrome *c* oxidase for facile purification of mutant forms. Biochem. J..

[27] Riistama S., Laakkonen L., Wikström M., Verkhovsky M.I., Puustinen A. (1999). The calcium binding site in cytochrome *aa*_3_ from *Paracoccus denitrificans*. Biochemistry.

[28] Maréchal A., Iwaki M., Rich P.R. (2013). Structural changes in cytochrome *c* oxidase induced by binding of sodium and calcium ions: an ATR-FTIR study. J. Am. Chem. Soc..

[29] Glasoe P.K., Long F.A. (1960). Use of glass electrodes to measure acidities in deuterium oxide. J. Phys. Chem..

[30] Rich P.R., Iwaki M. (2007). Methods to probe protein transitions with ATR infrared spectroscopy. Mol. BioSyst..

[31] Dodia R., Maréchal A., Bettini S., Iwaki M., Rich P.R. (2013). IR signatures of the metal centres of bovine cytochrome *c* oxidase: assignments and redox-linkage. Biochem. Soc. Trans..

[32] Maréchal A., Rich P.R. (2011). Water molecule reorganization in cytochrome *c* oxidase revealed by FTIR spectroscopy. Proc. Natl. Acad. Sci. U. S. A..

[33] Iwaki M., Puustinen A., Wikström M., Rich P.R. (2006). Structural and chemical changes of the P_M_ intermediate of *Paracoccus denitrificans* cytochrome *c* oxidase revealed by IR spectroscopy with labeled tyrosines and histidines. Biochemistry.

[34] Gorbikova E.A., Vuorilehto K., Wikström M., Verkhovsky M.I. (2006). Redox titration of all electron carriers of cytochrome *c* oxidase by Fourier transform infrared spectroscopy. Biochemistry.

[35] Rich P.R., Moody A.J., Gräber P., Milazzo G. (1997). Bioelectrochemistry: Principles and Practice.

[36] Qin L., Liu J., Mills D.A., Proshlyakov D.A., Hiser C., Ferguson-Miller S. (2009). Redox-dependent conformational changes in cytochrome *c* oxidase suggest a gating mechanism for proton uptake. Biochemistry.

[37] Liu J., Hiser C., Ferguson-Miller S. (2017). Role of conformational change and K-path ligands in controlling cytochrome *c* oxidase activity. Biochem. Soc. Trans..

[38] Iwaki M., Puustinen A., Wikström M., Rich P.R. (2003). ATR-FTIR spectroscopy of the P_M_ and F intermediates of bovine and *Paracoccus denitrificans* cytochrome *c* oxidase. Biochemistry.

[39] Lübben M., Gerwert K. (1996). Redox FTIR difference spectroscopy using caged electrons reveals contributions of carboxyl groups to the catalytic mechanism of haem-copper oxidases. FEBS Lett..

[40] Rich P.R., Maréchal A. (2008). Carboxyl group functions in the heme-copper oxidases: information from mid-IR vibrational spectroscopy. Biochim. Biophys. Acta.

[41] Artzatbanov V.Y., Konstantinov A.A., Skulachev V.P. (1978). Involvement of intramitochondrial protons in redox reactions of cytochrome a. FEBS Lett..

[42] Moody A.J., Rich P.R. (1989). Redox titration of haem *a* in cyanide-liganded cytochrome *c* oxidase: simulation studies on interacting, pH-dependent, redox centres. Biochem. Soc. Trans..

[43] Papa S. (2005). Role of cooperative H^+^/e• linkage (redox Bohr effect) at heme *a*/Cu_A_ and heme *a*_3_/Cu_B_ in the proton pump of cytochrome *c* oxidase. Biochem. Mosc..

[44] Maréchal A., Meunier B., Lee D., Orengo C., Rich P.R. (2012). Yeast cytochrome *c* oxidase: a model system to study mitochondrial forms of the haem-copper oxidase superfamily. Biochim. Biophys. Acta.

[45] Rich P.R., Breton J. (2001). FTIR studies of the CO and cyanide adducts of fully reduced bovine cytochrome *c* oxidase. Biochemistry.

[46] Rich P.R., Breton J. (2002). Attenuated total reflection Fourier transform infrared studies of redox changes in bovine cytochrome *c* oxidase: resolution of the redox Fourier transform infrared difference spectrum of heme *a*_3_. Biochemistry.

[47] Hellwig P., Behr J., Ostermeier C., Richter O.-M.H., Pfitzner U., Odenwald A., Ludwig B., Michel H., Mäntele W. (1998). Involvement of glutamic acid 278 in the redox reaction of the cytochrome *c* oxidase from *Paracoccus denitrificans* investigated by FTIR spectroscopy. Biochemistry.

[48] Lübben M., Prutsch A., Mamat B., Gerwert K. (1999). Electron transfer induces side-chain conformational changes of glutamate-286 from cytochrome *bo*_3_. Biochemistry.

[49] Nyquist R.M., Heitbrink D., Bolwien C., Wells T.A., Gennis R., Heberle J. (2001). Perfusion-induced redox differences in cytochrome *c* oxidase: ATR/FT-IR spectroscopy. FEBS Lett..

[50] Ferguson-Miller S., Hiser C., Liu J. (2012). Gating and regulation of the cytochrome *c* oxidase proton pump. Biochim. Biophys. Acta.

[51] Gorbikova E.A., Belevich N.P., Wikström M., Verkhovsky M.I. (2007). Protolytic reactions on reduction of cytochrome *c* oxidase studied by ATR-FTIR spectroscopy. Biochemistry.

[52] Heitbrink D., Sigurdson H., Bolwien C., Brzezinski P., Heberle J. (2002). Transient binding of CO to CuB in cytochrome *c* oxidase is dynamically linked to structural changes around a carboxyl group: a time-resolved step-scan Fourier transform infrared investigation. Biophys. J..

[53] Rost B., Behr J., Hellwig P., Richter O.M.H., Ludwig B., Michel H., Mäntele W. (1999). Time-resolved FT-IR studies on the CO adduct of *Paracoccus denitrificans* cytochrome *c* oxidase: comparison of the fully reduced and the mixed valence form. Biochemistry.

